# Synthesis of (*E*)-2-Styrylchromones and Flavones by Base-Catalyzed Cyclodehydration of the Appropriate β-Diketones Using Water as Solvent

**DOI:** 10.3390/molecules200611418

**Published:** 2015-06-22

**Authors:** Joana Pinto, Vera L. M. Silva, Ana M. G. Silva, Artur M. S. Silva

**Affiliations:** 1 Department of Chemistry & QOPNA, University of Aveiro, 3810-193 Aveiro, Portugal; E-Mail: jfpinto@ua.pt; 2 UCIBIO/REQUIMTE, Departamento de Química e Bioquímica, Faculdade de Ciências, Universidade do Porto, 4169-007 Porto, Portugal; E-Mail: ana.silva@fc.up.pt

**Keywords:** (*E*)-2-styrylchromones, flavones, cyclodehydration reactions, microwave heating, reactions in water

## Abstract

A low cost, safe, clean and environmentally benign base-catalyzed cyclodehydration of appropriate β-diketones affording (*E*)-2-styrylchromones and flavones in good yields is disclosed. Water was used as solvent and the reactions were heated using classical and microwave heating methods, under open and closed vessel conditions. β-Diketones having electron-donating and withdrawing substituents were used to evaluate the reaction scope. The reaction products were isolated in high purity by simple filtration and recrystallization from ethanol, when using 800 mg of the starting diketone under classical reflux heating conditions.

## 1. Introduction

**Figure 1:**
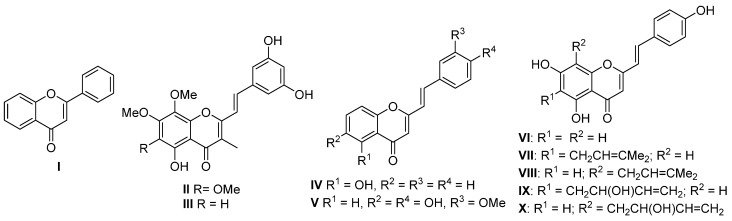
Structures of flavone **I** and naturally occurring (*E*)-2-styrylchromones **II**–**X**.

Flavones (**I**), the most prominent group of naturally occurring chromones, are present in a wide variety of plants [1] and are well-known by their broad range of biological properties, such as antibacterial, antifungal [2,3], antiviral [4], antiinflammatory [5], antioxidant [6], antiallergic [7], hepatoprotective [8], antithrombotic and antitumoral [9,10] activities (Figure 1). In contrast to flavones, there are only nine natural (*E*)-2-styrylchromones **II**–**X** [11,12,13,14,15], a group of oxygen heterocyclic compounds that although being scarce in Nature have shown significant biological activities [16,17,18] (Figure 1). 2-Styrylchromones have potential therapeutic applications in the treatment of cancer [19], allergies [20], viral infections [21], gout [22] and oxidative stress related damage [23]. These compounds have demonstrated strong protective effects against pro-oxidant agents observed in cellular [23] and in non-cellular systems [24], making them good antioxidant compound candidates.

**Scheme 1:**
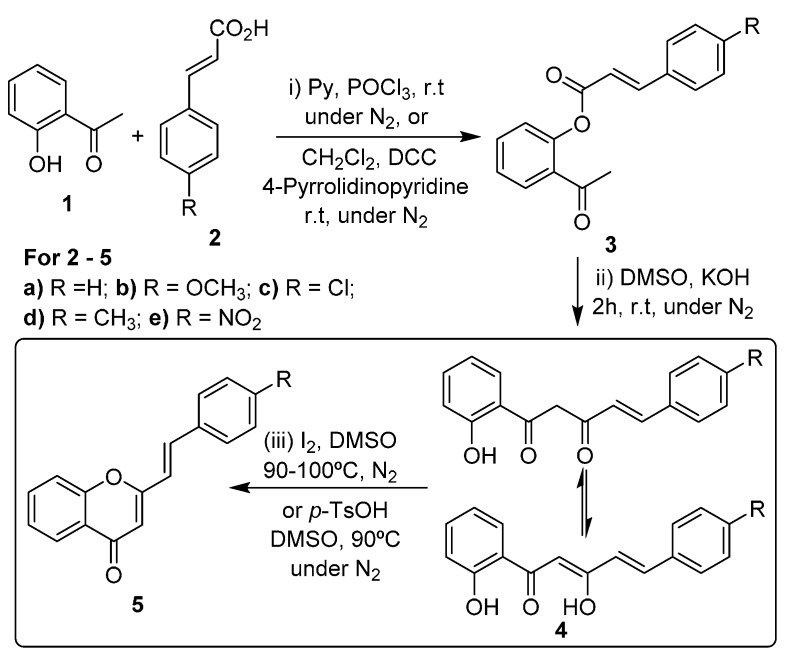
Synthesis of (*E*)-2-styrylchromones by the Baker-Venkataraman method.

The most common strategies for the synthesis of (*E*)-2-styrylchromones are the aldol condensation of 2′-hydroxyacetophenone derivatives with cinnamaldehydes, followed by cyclodehydrogenation of the formed 2′-hydroxycinnamylideneacetophenones, and the Baker-Venkataraman method [16,17,18,25]. This second strategy involves the *O*-acylation of 2′-hydroxyacetophenone (**1**, Scheme 1, step i) followed by base-catalyzed rearrangement of the formed esters **3** to give 5-aryl-1-(2-hydroxyphenyl)pent-4-ene-1,3-diones **4** (which exist in equilibrium with their enolic form; Scheme 1, step ii). This rearrangement can be performed in high yield under solvent-free conditions, using grinding techniques [26]. The last step of this method consists in the cyclodehydration of compounds **4** into (*E*)-2-styrylchromones **5**, using strong acidic conditions, *p*-toluenesulfonic acid or a catalytic amount of iodine in DMSO at 90–100 °C (Scheme 1, step iii) [16,17,18,25].

A similar procedure is also well-established for the synthesis of flavones. Also in this case the cyclodehydration reaction of 1-(2-hydroxyphenyl)propane-1,3-diones (which exist also in equilibrium with their enolic forms) is an important step in the synthesis of flavones using the Baker-Venkataraman approach (the same sequence presented in Scheme 1, but using benzoic acids instead of cinnamic acids) [27,28,29]. This process usually is a catalytic transformation performed in different media. Some reaction conditions employed an excess of sulfuric acid in glacial acetic acid [30], cationic exchange resins in isopropyl alcohol [31], CuCl_2_ in ethanol [32], ionic liquids under microwave irradiation [33], heteropolyacids, carbon supported triflic acid [34,35,36,37] and also grinding techniques in the presence of phosphorus pentoxide [38]. Some of these methods require high temperatures or long times to complete the reactions [30,31,37], other require the preparation of the catalysts, involving non-green and time consuming procedures [34,35,36,37], and almost all of them were used to prepare flavones or simple chromones, but were not used in the synthesis of 2-styrylchromones [30,31,32,33,34,35,36,37]. Pawar and co-workers [33] described the conversion of 1-(2-hydroxyphenyl)-3-phenylpropane-1,3-diones to the corresponding flavones under microwave irradiation using the ionic liquid [EtNH_3_]NO_3_·(EAN). Despite the structural differences, EAN shares many properties with water [39]. However, while water is a safe, easily available and relatively low cost and environmentally friendly solvent, the ionic liquid has to be prepared. EAN is usually synthesized by heating ethyl nitrate with an alcoholic solution of ammonia or by reacting ethylamine with concentrated nitric acid [40] which cannot be considered green processes. In addition organic nitrates are potentially explosive, especially when rigorously dried. The recovery of the ionic liquid requires evaporation of the aqueous layer at 80 °C that represents additional time and energy consumption.

The synthesis of flavones using trifluoromethanesulfonic acid supported on carbon as catalyst [37] was performed using toluene as solvent and requires 5 h for reaction completion. Makrandi and co-workers [38] described a green synthesis of flavones and 2-styrylchromones via cyclodehydration of the corresponding 1-(2-hydroxyaryl)-3-(aryl/styryl)propane-1,3-diones under solvent-free conditions in the presence of phosphorus pentoxide using grinding techniques. However the scope of the reaction was poorly checked since only substrates containing electron-donating substituents were used.

A different and eco-friendly methodology for the direct synthesis of flavones, from phloroglucinol and β-ketoesters, was reported by Seijas and co-workers [41]. The reaction involves the cycloaddition of an α-oxo ketene intermediate followed by an uncatalyzed thermal Fries rearrangement. The flavones were obtained in very good yields (68%–96%) after 3 min of microwave irradiation using solvent-free conditions, but the methodology was not extended to the synthesis of 2-styrylchromones. Due to the formation of polar transition states, the reaction benefits from microwave activation, however 800 W output power and 240 °C are required to achieve short reaction times and high reaction yields. Furthermore, when the melting point of the reactants is higher a longer reaction time is required.

**Scheme 2:**
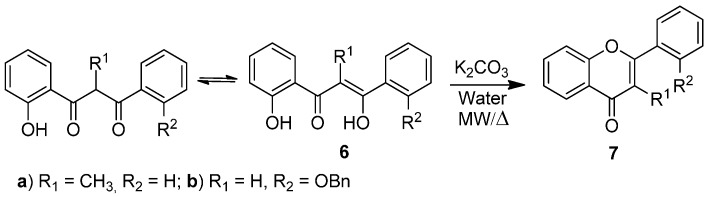
Cyclodehydration reaction of 3-aryl-1-(2-hydroxyphenyl)propane-1,3-diones **6a**,**b** to flavones **7a**,**b**.

Our interest in organic reactions using exclusively water as solvent prompted us to investigate the aqueous cyclodehydration of appropriate β-diketones to prepare (*E*)-2-styrylchromones and flavones. The use of water in organic synthesis, without the presence of any organic solvent, can be beneficial because water is an available, cheap, safe and environmentally benign solvent. So far, extensive work revealed that a variety of organic reactions including dehydration reactions can be performed using water as solvent [42,43,44]. Here we present a protocol for the base-catalyzed cyclodehydration reaction of compounds **4** and **6** into the corresponding (*E*)-2-styrylchromones **5** and flavones **7** in water, in the absence of any organic solvent, and using potassium carbonate as base (Scheme 1 and Scheme 2). The desired compounds **5** and **7** were obtained in yields over 60%, except for compound **7b**. This method represents a cheap, easy and suitable protocol for the cyclodehydration of 5-aryl-1-(2-hydroxyphenyl)pent-4-ene-1,3-diones **4** and 3-aryl-1-(2-hydroxyphenyl)propane-1,3-diones **6** (which exist in equilibrium with their enolic forms) which contributes to the establishment of environmentally friendly organic synthesis.

## 2. Results and Discussion

The typical established methods for the cyclodehydration reaction of 5-aryl-1-(2-hydroxyphenyl)pent-4-ene-1,3-diones **4** involve the use of strong acidic conditions, *p*-toluenesulfonic acid, or a catalytic amount of iodine in DMSO at 90–100 °C [16,17,18,25]. In order to establish a more environmental friendly procedure for this cyclodehydration reaction commonly used in our research group, we performed the base-catalyzed (potassium carbonate) cyclodehydration reaction of **4a** in water. To the best of our knowledge, the cyclodehydration reaction of 5-aryl-1-(2-hydroxyphenyl)pent-4-ene-1,3-diones **4** in aqueous basic conditions has not been previously reported. Firstly, we performed the reactions in a small scale (50–200 mg), in order to establish the optimal reaction conditions and to fully characterize the obtained compounds.

**Scheme 3:**
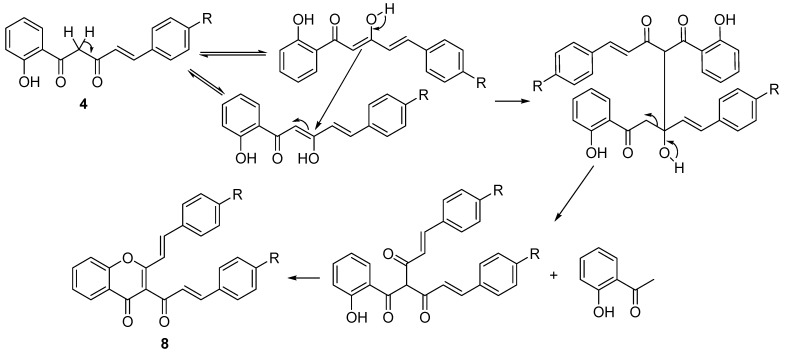
Proposed mechanism for the formation of (*E,E*)-3-cinnamoyl-2-styrylchromones **8**.

Our results indicate that 0.5 molar equiv of base are enough to perform the expected cyclodehydration reaction under classical reflux heating (Table 1, Entries 1 and 2). Under these conditions (*E*)-2-styrylchromone **5a** was obtained in 59% yield. In order to increase the yield, and since the used β-diketone is not soluble in water, we tried the addition of a phase transfer catalyst (PTC) (tetrabutylammonium bromide, TBAB) to improve the solubility of **4a**. However in the presence of this catalyst the yield was only 12% (Table 1, Entry 3). We also tried to replace potassium carbonate by another base, tetramethylammonium hydroxide (TMAOH) as a bifunctional catalyst, acting as base and as PTC (Table 1, Entries 4 and 5). Since the product was obtained in low yields, we concluded that the reaction works better in the first conditions, using potassium carbonate as base. Under these conditions (*E*)-2-styrylchromones **5b**–**e** were obtained as main compounds in 61%, 70%, 70% and 20% yields, respectively (Table 1, Entries 6–9) and two by-products were also identified in each case. The NMR spectra of the by-product with higher Rf value, obtained in very low yield (less than 10%), is consistent with a benzalacetone structure, resulting from the hydrolysis of the starting material **4** in alkaline medium [45]. The other by-product with lower Rf value was identified as the (*E,E*)-3-cinnamoyl-2-styrylchromone **8** (see NMR spectra in Supporting Information, Figure S14) and was obtained in very low yield (less than 5%). A plausible mechanism for the formation of this compound is similar to the mechanism proposed for the formation of the (*E,E*)-3-cinnamoyl-2-styrylchromone in the cyclodehydration of the corresponding 5-aryl-3-hydroxy-1-(2-hydroxyaryl)-2,4-pentadien-1-one, when performed with a mixture of DMSO and a catalytic amount of iodine at 90 °C [46] (Scheme 3).

**Table 1:** Cyclodehydration of 5-aryl-1-(2-hydroxyphenyl)pent-4-ene-1,3-diones **4a**–**e** to (*E*)-2-styrylchromones **5a**–**e** in water, using classical reflux heating conditions.

Entry	Compound	Base/molar equiv	Time (h)	Yield of 5 (%) ^a^
1	**4a** R = H	K_2_CO_3_/1	4 ^b^	58
2	**4a** R = H	K_2_CO_3_/0.5	4	59
3	**4a** R = H	K_2_CO_3_/0.5 + TBAB/0.5	3	12
4	**4a** R = H	TMAOH/1	2	19
5	**4a** R = H	TMAOH/0.5	2	47
6	**4b** R = OCH_3_	K_2_CO_3_/0.5	4	61
7	**4c** R = Cl	K_2_CO_3_/0.5	4	70
8	**4d** R = CH_3_	K_2_CO_3_/0.5	4 ^b^	70
9	**4e** R = NO_2_	K_2_CO_3_/0.5	4	20
10	**4a** R = H	K_2_CO_3_/0.5	4	55 ^c^

^a^ Yields obtained after purification of compounds by thin layer chromatography; ^b^ Reaction was almost complete after 2 h; ^c^ Cyclodehydration reaction of **4a** was performed on a bigger scale (800 mg). The yield was determined after filtration of the product precipitate and recrystallization.

In order to isolate the product by simple filtration, avoiding the use of the expensive and time-consuming chromatographic techniques, the cyclodehydration of **4a** was performed on a bigger scale (800 mg) thus establishing an easy scalable protocol. In this case, the yield obtained of **5a** was 55%, which was determined through precipitation (after adjustment of pH to 3–4), filtration and recrystallization of the product **5a** (Table 1, Entry 10). Since 2-styrylchromones **5a**–**e** are water insoluble the scalability of the reaction facilitates the precipitation and product isolation.

To evaluate the effect of microwave radiation in this reaction we performed the base-catalyzed cyclodehydration reaction of 1-(2-hydroxyphenyl)-5-phenylpent-4-ene-1,3-dione **4a** under open and closed vessel conditions, using water as solvent. Water is a good solvent for microwave assisted synthesis due to its high dielectric constant [47]. So, we studied the cyclodehydration reaction of **4a** under different temperatures, reaction time and amount of base (results summarized in Table 2). Using open vessel conditions (reflux, 30 min), the yields for (*E*)-2-styrylchromones **5a**,**b**,**d** were 66%, 56% and 55%, respectively (Table 2, Entries 3, 5 and 9), but this reaction time is not suitable for the synthesis of (*E*)-2-styrylchromones with electron-accepting substituents such as **5c**,**e** (Table 2, Entries 7 and 11). In these cases better yields were obtained (58% and 56%, respectively) after 10 min of irradiation (Table 2, Entries 6 and 10).

**Table 2:** Cyclodehydration of 5-aryl-1-(2-hydroxyphenyl)pent-4-ene-1,3-diones **4a**–**e** to (*E*)-2-styrylchromones **5a**–**e** in water, under microwave irradiation, using open vessel reflux and closed vessel (120 °C) conditions.

Entry	Compound	Method	Base/molar equiv	Time (min)	Yield of 5 (%) ^a^
1	**4a** R =H	Open	K_2_CO_3_/1	10	35
2	**4a** R =H	Open	K_2_CO_3_/0.5	5	54
3	**4a** R = H	Open	K_2_CO_3_/0.5	30	66
4	**4b** R = OCH_3_	Open	K_2_CO_3_/0.5	10	47
5	**4b** R = OCH_3_	Open	K_2_CO_3_/0.5	30	56
6	**4c** R = Cl	Open	K_2_CO_3_/0.5	10	58
7	**4c** R = Cl	Open	K_2_CO_3_/0.5	30	47
8	**4d** R = CH_3_	Open	K_2_CO_3_/0.5	10	45
9	**4d** R = CH_3_	Open	K_2_CO_3_/0.5	30	55
10	**4e** R = NO_2_	Open	K_2_CO_3_/0.5	10	56
11	**4e** R = NO_2_	Open	K_2_CO_3_/0.5	30	38
12	**4a** R =H	Closed	K_2_CO_3_/0.5	5	50
13	**4a** R = H	Closed	K_2_CO_3_/0.5	30	67
14	**4b** R = OCH_3_	Closed	K_2_CO_3_/0.5	30	77
15	**4c** R = Cl	Closed	K_2_CO_3_/0.5	10	66
16	**4c** R = Cl	Closed	K_2_CO_3_/0.5	15	38
17	**4c** R = Cl	Closed	K_2_CO_3_/0.5	30	41
18	**4d** R = CH_3_	Closed	K_2_CO_3_/0.5	30	57
19	**4e** R = NO_2_	Closed	K_2_CO_3_/0.5	30	27
20	**4e** R = NO_2_	Closed	TMAOH/0.5	10	60

^a^ Yields obtained after purification of compounds by thin layer chromatography.

Employing closed vessel conditions (120 °C, 30 min) compounds **5a**,**b**,**d** were obtained in 67%, 77% and 57% yields, respectively (Table 2, Entries 13, 14 and 18). For compounds **5c**,**e** we observed a high degradation in the reaction mixture after 30 min of closed vessel microwave conditions (Table 2, Entries 17 and 19), but good yields (66% and 60%, respectively) were obtained only with 10 min of irradiation (Table 2, Entries 15 and 20). In the case of the cyclodehydration of derivative **4e**, TMAOH was used as base and as PTC in order to improve the solubility of this compound in water which led to the formation of compound **5e** in 60% yield (Table 2, Entry 20).

We also performed the reaction with a lower amount of K_2_CO_3_ (0.05 equiv) using closed vessel microwave conditions. In a first attempt the cyclodehydration of **4a** was performed at 120 °C for 30 min; however the 2-styrylchromone **5a** was obtained in 16% yield and 26% of **4a** was recovered. In order to improve the yield, we repeated the reaction at 200 °C (for 30 min). Under these conditions, compounds **5a**–**e** were obtained in 64%–75% yield (Table 3).

**Table 3:** Cyclodehydration of 5-aryl-1-(2-hydroxyphenyl)pent-4-ene-1,3-diones **4a**–**e** to (*E*)-2-styrylchromones **5a**–**e** in water and in the presence of a catalytic amount of K_2_CO_3_, using closed vessel microwave conditions, at 200 °C.

Entry	Compound	Base/molar equiv	Time (min)	Yield of 5 (%) ^a^
1	**4a** R = H	K_2_CO_3_/0.05	30	65
2	**4b** R = OCH_3_	K_2_CO_3_/0.05	30	75
3	**4c** R = Cl	K_2_CO_3_/0.05	30	64
4	**4d** R = CH_3_	K_2_CO_3_/0.05	30	68
5	**4e** R = NO_2_	K_2_CO_3_/0.05	30	69

^a^ Yields obtained after purification of compounds by thin layer chromatography.

Comparing the results obtained in classical and microwave heating methods, a significant reduction of the reaction time was achieved from 2–4 h (classical heating) to 10–30 min (microwave heating). In addition, we demonstrated that it is possible to use a catalytic amount of base (0.05 equiv) using rapid microwave heating at a substantially higher temperature (at 200 °C), taking advantage of the use of water under closed vessel microwave conditions.

Recently, a new synthetic route of flavones was reported, consisting in a one-pot procedure by treatment of 2′-hydroxyacetophenones with 3 molar equiv of aroyl chloride in net K_2_CO_3_/acetone (1% w/w water) [48]. Under these conditions flavones were obtained in 51%–65% yields together with 3-aroylflavones (11%–23%); this study was not extended to the synthesis of 2-styrylchromones.

After establishing the best conditions for the base-catalyzed cyclodehydration reaction of 5-aryl-1-(2-hydroxyphenyl)pent-4-ene-1,3-diones **4a**–**e** under classical reflux heating and microwave irradiation, we extended our study to other 3-aryl-1-(2-hydroxyphenyl)propane-1,3-diones **6a**,**b** in order to prepare flavones **7a**,**b** (Scheme 2, Table 4). Cyclodehydration of compound **6a** occurs in excellent yield (quantitative yield) in both classical reflux heating for 1 h and under microwave irradiation for 30 min (Table 4, Entries 1 and 3). In the case of compound **6b** the reaction was performed only under closed vessel microwave conditions. After 15 min of irradiation some unreacted starting material was observed while after 30 min the expected flavone **7b** was obtained in 45% yield (Table 4, Entry 4). However after longer reaction time (45 min) the yield was not improved (46% yield) due to high degradation in the reaction mixture (Table 4, Entry 5). These results indicate that the substituents on the diketone structure have a great effect on the yield of the cyclodehydration reaction.

**Table 4:** Cyclodehydration reaction of 3-aryl-1-(2-hydroxyphenyl)propane-1,3-diones **6a**,**b** into flavones **7a**,**b**, under classical reflux heating and closed vessel microwave conditions, at 200 °C.

Entry	Compound	Method	Reaction Time (min)	Yield of 7 (%) ^a^
1	**6a** R^1^ =CH_3_, R^2^ = H	Oil bath	60	Quantitative
2	**6a** R^1^ =CH_3_, R^2^ = H	Microwave	15	70
3	**6a** R^1^ =CH_3_, R^2^ = H	Microwave	30	Quantitative
4	**6b** R^1^ =H, R^2^ = OBn	Microwave	30	45
5	**6b** R^1^ =H, R^2^ = OBn	Microwave	45	46

^a^ Yields obtained after purification of compounds by thin layer chromatography.

**Scheme 4:**
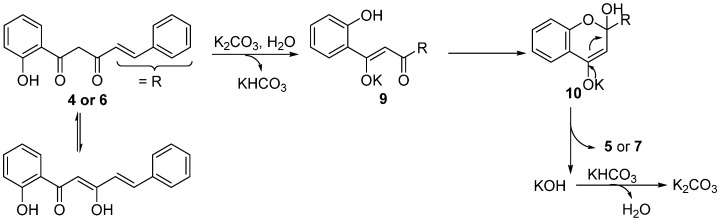
Possible mechanism on base-catalyzed cyclodehydration of β-diketones **4a**–**e** and **6a**,**b** in water.

A plausible mechanism for the formation of 2-styrylchromones **5a**–**e** and flavones **7a**,**b** under the described experimental conditions is proposed in Scheme 4. Enolization of the α-carbonyl group in **4** or **6**, leads to intermediate **9** in the presence of a base (K_2_CO_3_), and intramolecular cycloaddition of **9** gives **10**. Rearomatization of **10** by elimination of KOH provides the corresponding products **5** or **7**. A similar mechanism was proposed by Fu and coworkers for the synthesis of chromones by K_2_CO_3_-catalysed (0.20 equiv) intramolecular cyclization of the corresponding diketones in DMF [49].

## 3. Experimental Section

### 3.1. General Information

Melting points were determined on a Büchi B-545 melting point apparatus. Preparative thin-layer chromatography when used was carried out with Merck silica gel (60 DGF254). NMR spectra were recorded on a Bruker Avance 300 spectrometer (300.13 MHz for ^1^H and 75.47 MHz for ^13^C), with CDCl_3_ used as solvent if not stated otherwise. The internal standard was TMS (see Supporting Information). Microwave-assisted reactions were carried out in a CEM Discovery Labmate circular single-mode cavity instrument (300 W max magnetron power output) from CEM Corporation. Reactions carried out in open vessel were performed using a 50 mL round-bottom flask filled with the reaction mixture and equipped with a condensator under nitrogen atmosphere. Reactions carried out in closed vessel were performed in a sealed 10 mL vessel and 100 psi of pressure was defined as the limit to operate in safe conditions. In open and closed vessel conditions the temperature was always controlled using the vertically focused IR temperature sensor (40–300 °C temperature range) of the CEM monomode microwave, for volume-independent non-invasive temperature measurement.

### 3.2. Optimized Experimental Procedure for the Cyclodehydration Reaction of β-Diketones **4a**–**e** and **6a** under Classical Heating Conditions

Potassium carbonate (52.52 mg, 0.38 mmol) was added to the appropriate β-diketone **4a**–**e** or **6a** (0.75 mmol) in distilled water (10–15 mL). The mixture was refluxed under nitrogen atmosphere for (2–4 h, see Table 1) until the consumption of starting material (the reaction progress was monitored by tlc). After that period, the mixture was acidified at pH 3–4 with a 20% HCl solution and the obtained product extracted with ethyl acetate (3 × 50 mL). The combined organic layer was dried with anhydrous sodium sulfate and concentrated under reduced pressure. In some cases the obtained compound was purified by thin layer chromatography using dichloromethane as eluent. When the reaction was performed in a bigger scale (3.0 mmol) the product precipitates after acidification at pH 3–4 and was isolated by filtration and recrystallized from ethanol. Compounds were obtained as yellow solids (**5a**, 109.9 mg, 59%; **5b**, 127.3 mg, 61%; **5c**, 148.4 mg, 70%; **5d**, 137.7 mg, 70%; **5e**, 44.0 mg, 20%), or white solids (**7a**, 177.2 mg, quantitative yield).

### 3.3. Optimized Experimental Procedure for the Cyclodehydration Reaction of β-Diketones **4a**–**e** under Microwave Irradiation in Open Vessels

Potassium carbonate (52.52 mg, 0.38 mmol) was added to the appropriate β-diketone **4a**–**e** (0.75 mmol) in distilled water (7 mL). The mixture was refluxed under microwave irradiation using a 50 mL round-bottom flask equipped with a condenser under nitrogen atmosphere (for reaction time see Table 2). After that period, the mixture was acidified at pH 3–4 with a 20% HCl solution and the product was extracted with ethyl acetate (3 × 50 mL). The combined organic layer was dried with anhydrous sodium sulfate and concentrated under reduced pressure. In some cases the obtained compound was purified by thin layer chromatography using dichloromethane as eluent. Compounds were obtained as yellow solids (**5a**, 122.9 mg, 66%; **5b**, 116.9 mg, 56%; **5c**, 123.0 mg, 58%; **5d**, 108.2 mg, 55%; **5e**, 123.2 mg, 56%).

### 3.4. Optimized Experimental Procedure for the Cyclodehydration Reaction of β-Diketones **4a**–**e** and **6a**,**b** under Microwave Irradiation in Closed Vessels

The appropriate amount of potassium carbonate (0.5 equiv or 0.05 equiv) was added to the appropriate β-diketone **4a**–**e** or **6a**,**b** (0.19 mmol) in distilled water (3.5 mL). The mixture was heated at 120 °C or 200 °C in a 10 mL closed vessel under microwave irradiation (for reaction time see Table 2, Table 3 and Table 4). After that period, the mixture was acidified at pH 3–4 with a 20% HCl solution and the product was extracted with ethyl acetate (3 × 50 mL). The combined organic layer was dried with anhydrous sodium sulfate and concentrated under reduced pressure. In some cases the obtained compound was purified by thin layer chromatography using dichloromethane as eluent. Compounds were obtained as yellow solids (at 120 °C: **5a**, 31.6 mg, 67%; **5b**, 40.7 mg, 77%; **5c**, 35.4 mg, 66%; **5d**, 28.4 mg, 57%; **5e**, 33.4 mg, 60%. At 200 °C: **5a**, 30.7 mg, 65%; **5b**, 39.7 mg, 75%; **5c**, 34.4 mg, 64%; **5d**, 33.9 mg, 68%; **5e**, 38.4 mg, 69%) or white solids (**7a**, 44.9 mg, quantitative yield; **7b**, 28.7 mg, 46%). The melting points of compounds **5a**–**e** and **7a**,**b** were determined and compared with those reported in the literature [50,51,52,53] (Table 5 and Table 6). Due to the difference between the measured melting points and those from the literature for compounds **5b** and **7a**, 3 samples of each one of these compounds, obtained in three different experiments, were taken, analyzed by NMR and the corresponding melting points were determined. All the samples were dried on a vacuum line. For all the three experiments the structure and purity of compounds **5b** and **7a** was confirmed by NMR. The experimental melting points were always around 125–126 °C for **5a** and 73–74 °C for **7a** and very different from that reported on the literature 139–140 °C and 110–112 °C, respectively.

**Table 5:** Yield and melting point data of the compounds **5a**–**e**.

Compounds	R	Yield	Melting Point (°C)	Melting Point (°C) (Lit.) [50,51]
**5a**	H	67	136–137	133–134
**5b**	OCH_3_	77	125–126	139–140 ^a^
**5c**	Cl	70	218–219	224–226
**5d**	CH_3_	70	158–159	159–160
**5e**	NO_2_	60	276–278	282–283

^a^ This compound was synthesized and characterized three times. The NMR data proved the structure and the determined melting point was the same in the three determinations.

**Table 6:** Yield and melting point data of the compounds **7a**,**b**.

Compounds	R^1^	R^2^	Yield	Melting Point (°C)	Melting Point (°C) (Lit.)
**7a**	CH_3_	H	Quant.	73–74	110–112 ^a^ [52]
**7b**	H	OBn	46	82–84	82–83 [53]

^a^ This compound was synthesized and characterized three times. The NMR data proved the structure and the determined melting point was the same in the three determinations.

## 4. Conclusions

A new methodology for the cyclodehydration reaction of the appropriate β-diketones to prepare a variety of (*E*)-2-styrylchromones and flavones in good yields (67%–75%) and (46% to quantitative yield), respectively, was established. Comparing to the yields obtained using the usual established methods (69%–94%) [47,48], our yields are similar as well as the compounds’ purity. The use of water as a solvent, offers significant environmental advantages and facilitates the isolation and purification of the products when compared with the DMSO, which is the solvent typically use in such reactions. Furthermore, this new methodology should allow the easy scale up of the reaction being cheaper, safe and more environmentally friendly than the traditional methods.

## Supplementary Materials

^1^H- and ^13^C-NMR spectra of compounds **5a**–**e** (Figures S1–S9) and **7a**,**b** (Figures S10–S13) and ^1^H-NMR spectrum of compound **8** (Figure S14). Supplementary materials can be accessed at: http://www.mdpi.com/1420-3049/20/6/11418/s1.
