# Supplementary material for: Synthesis of (E)-2-Styrylchromones and Flavones by Base-Catalyzed Cyclodehydration of the Appropriate β-Diketones Using Water as Solvent
Source: Molecules. 2015 Jun 22;20(6):11418–31. doi: 10.3390/molecules200611418 (PMC6272435; doi:10.3390/molecules200611418)
Supplement: Supplementary file 1 [file molecules-20-11418-s001.pdf]

Supplementary Material

Table of Contents

**NMR Spectra**	S2
**Figure S1.** ^{1}H-NMR spectrum of (*E*)-2-styrylchromone **5a** ($CDCl_3$, 300.13 MHz)	S2
**Figure S2.** ^{13}C-NMR spectrum of (*E*)-2-styrylchromone **5a** ($CDCl_3$, 75.47 MHz)	S3
**Figure S3.** ^{1}H-NMR spectrum of (*E*)-2-[2-(4-methoxyphenyl)vinyl]-4*H*-chromen-4-one **5b** ($CDCl_3$, 300.13MHz)	S3–S4
**Figure S4.** ^{13}C-NMR spectrum of (*E*)-2-[2-(4-methoxyphenyl)vinyl]-4*H*-chromen-4-one **5b** ($CDCl_3$, 75.47 MHz)	S4
**Figure S5.** ^{1}H-NMR spectrum of (*E*)-2-[2-(4-chlorophenyl)vinyl]-4*H*-chromen-4-one **5c** ($CDCl_3$, 300.13 MHz)	S5
**Figure S6.** ^{13}C-NMR spectrum of (*E*)-2-[2-(4-chlorophenyl)vinyl]-4*H*-chromen-4-one **5c** ($CDCl_3$, 75.47 MHz)	S6
**Figure S7.** ^{1}H-NMR spectrum of (*E*)-2-[2-(4-methylphenyl)vinyl]-4*H*-chromen-4-one **5d** ($CDCl_3$, 300.13 MHz)	S6–S7
**Figure S8.** ^{13}C-NMR spectrum of (*E*)-2-[2-(4-methylphenyl)vinyl]-4*H*-chromen-4-one **5d** ($CDCl_3$, 75.47 MHz)	S7
**Figure S9.** ^{1}H-NMR spectrum of (*E*)-2-[2-(4-nitrophenyl)vinyl]-4*H*-chromen-4-one **5e** (DMSO-d_6, 300.13 MHz)	S8
**Figure S10.** ^{1}H-NMR spectrum of 3-methylflavone **7a** ($CDCl_3$, 300.13 MHz)	S9
**Figure S11.** ^{13}C-NMR spectrum of 3-methylflavone **7a** ($CDCl_3$, 75.47 MHz)	S9
**Figure S12.** ^{1}H-NMR spectrum of 2′-benzyloxyflavone **7b** ($CDCl_3$, 300.13 MHz)	S10
**Figure S13.** ^{13}C-NMR spectrum of 2′-benzyloxyflavone **7b** ($CDCl_3$, 75.47 MHz)	S11
**Figure S14.** ^{1}H-NMR spectrum of 3-cinnamoyl-2-((*E*)-styryl)-4*H*-chromen-4-one **8** ($CDCl_3$, 300.13 MHz)	S11–S12

NMR Spectra

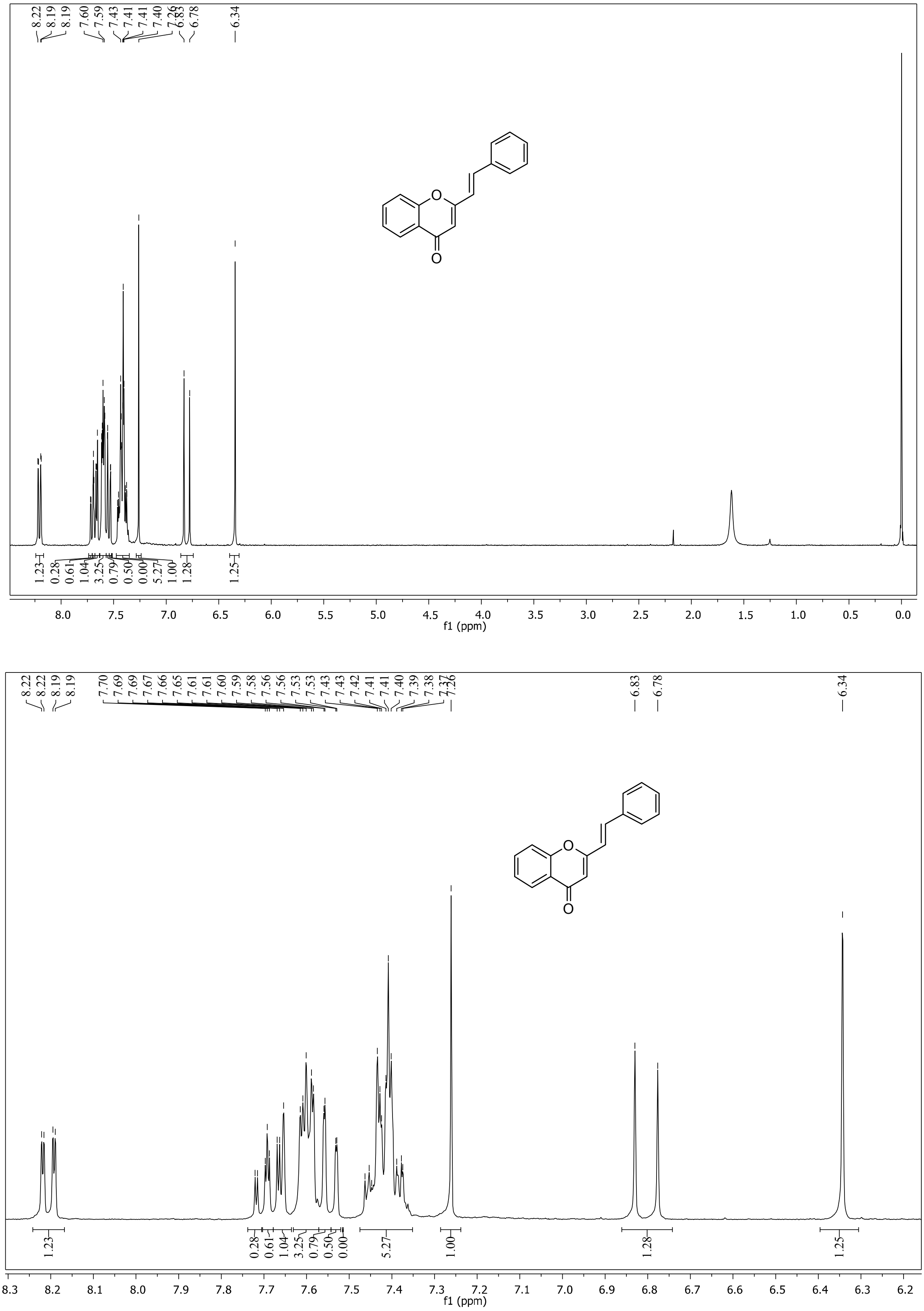

Figure S1. ^{1}H-NMR spectrum of (*E*)-2-styrylchromone **5a** ($CDCl_3$, 300.13 MHz).

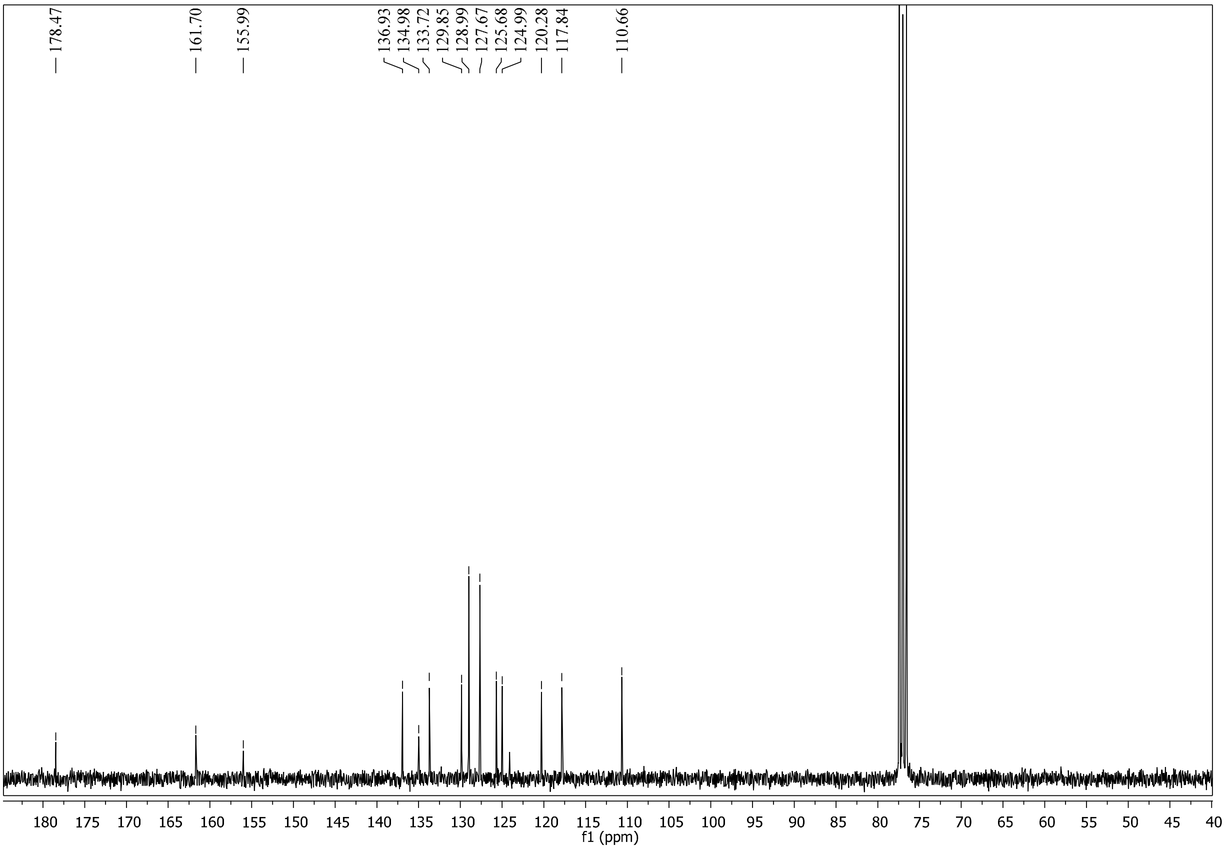

Figure S2. ^{13}C-NMR spectrum of (*E*)-2-styrylchromone **5a** ($CDCl_3$, 75.47 MHz).

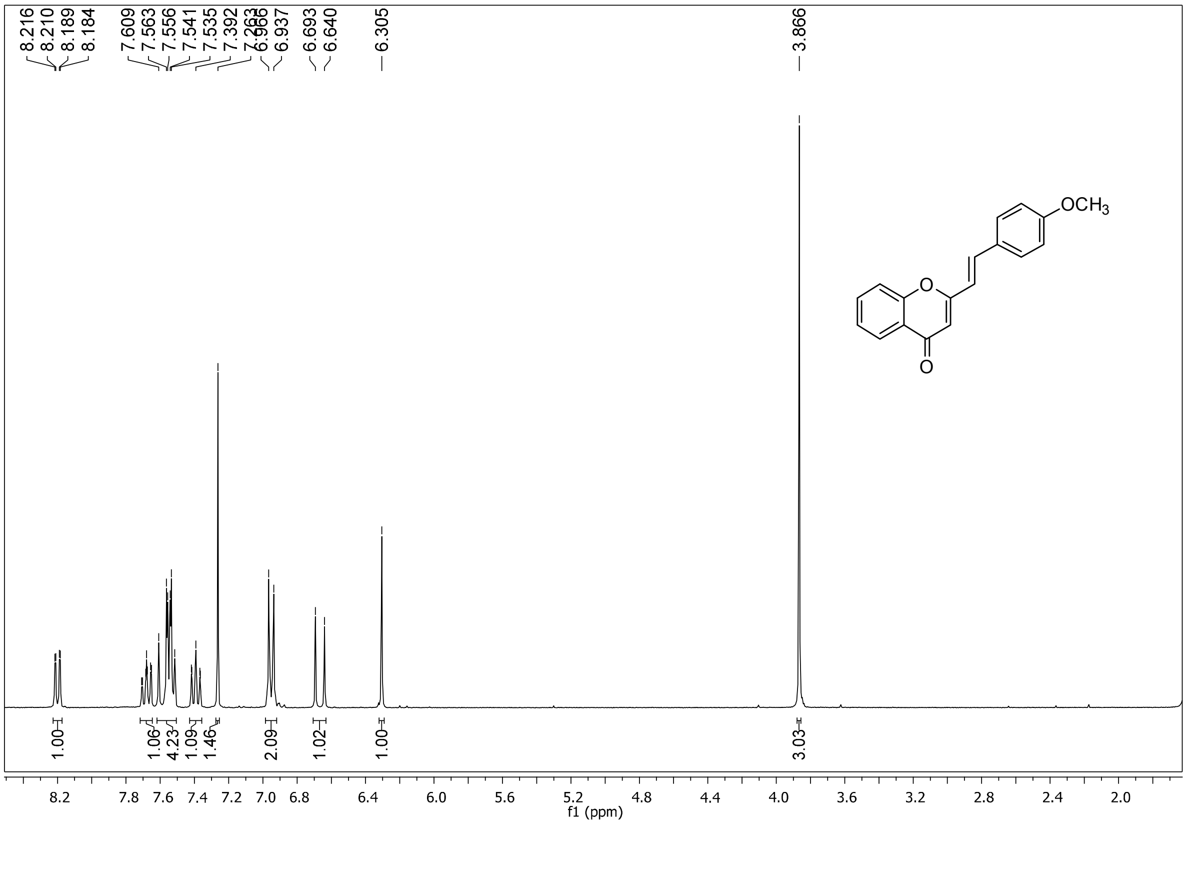

Figure S3. *Cont.*

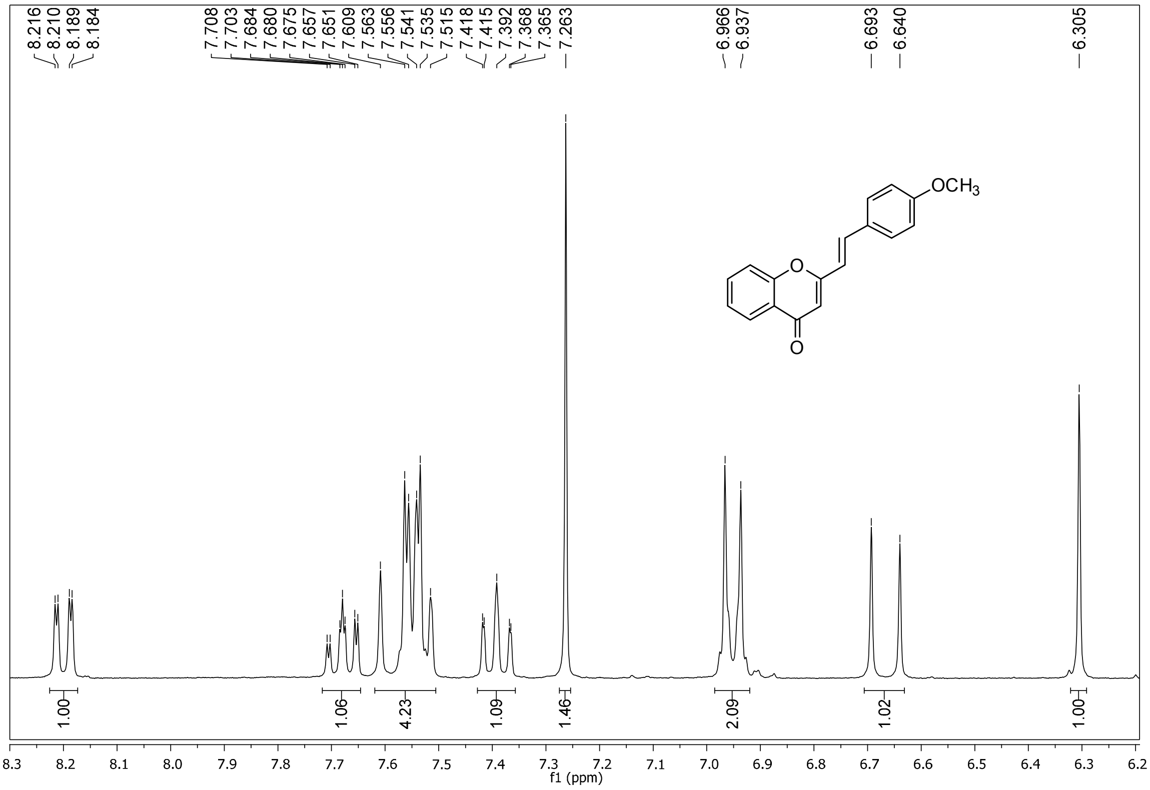

Figure S3. ^{1}H-NMR spectrum of (*E*)-2-[2-(4-methoxyphenyl)vinyl]-4*H*-chromen-4-one **5b** ($CDCl_3$, 300.13 MHz).

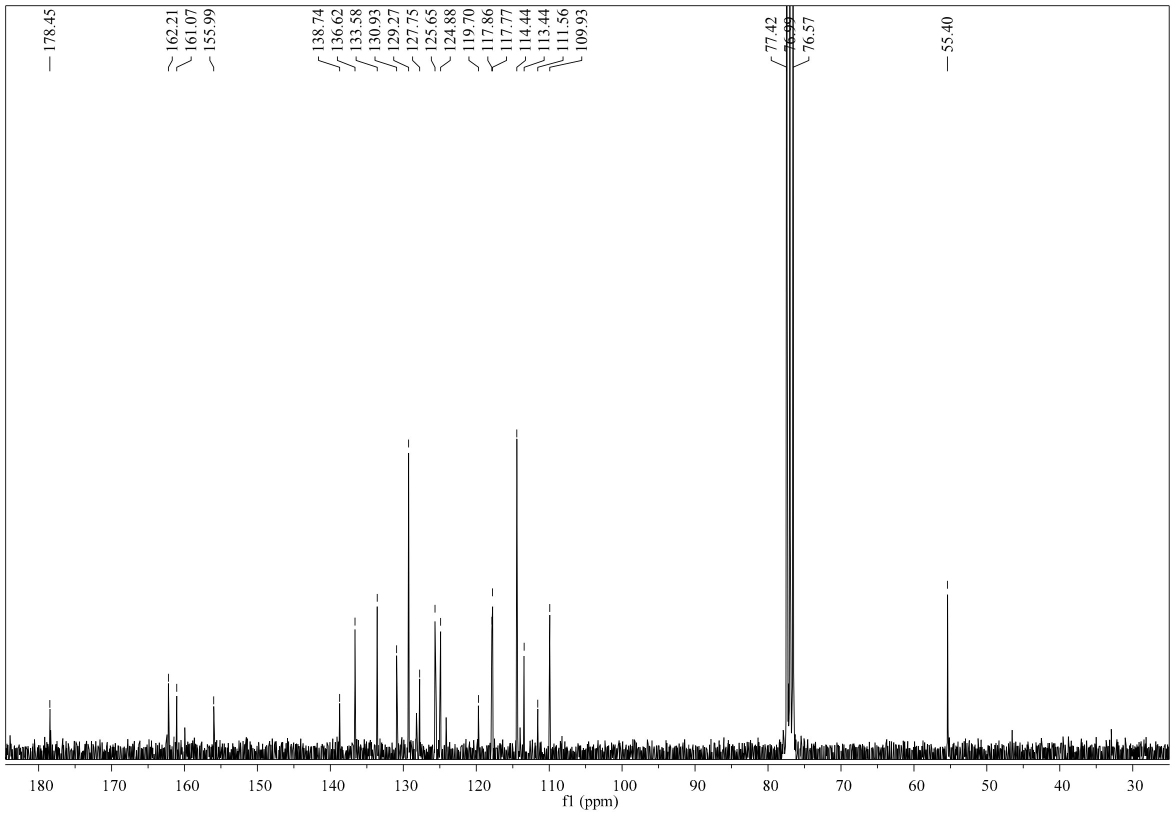

Figure S4. ^{13}C-NMR spectrum of (*E*)-2-[2-(4-methoxyphenyl)vinyl]-4*H*-chromen-4-one **5b** ($CDCl_3$, 75.47 MHz).

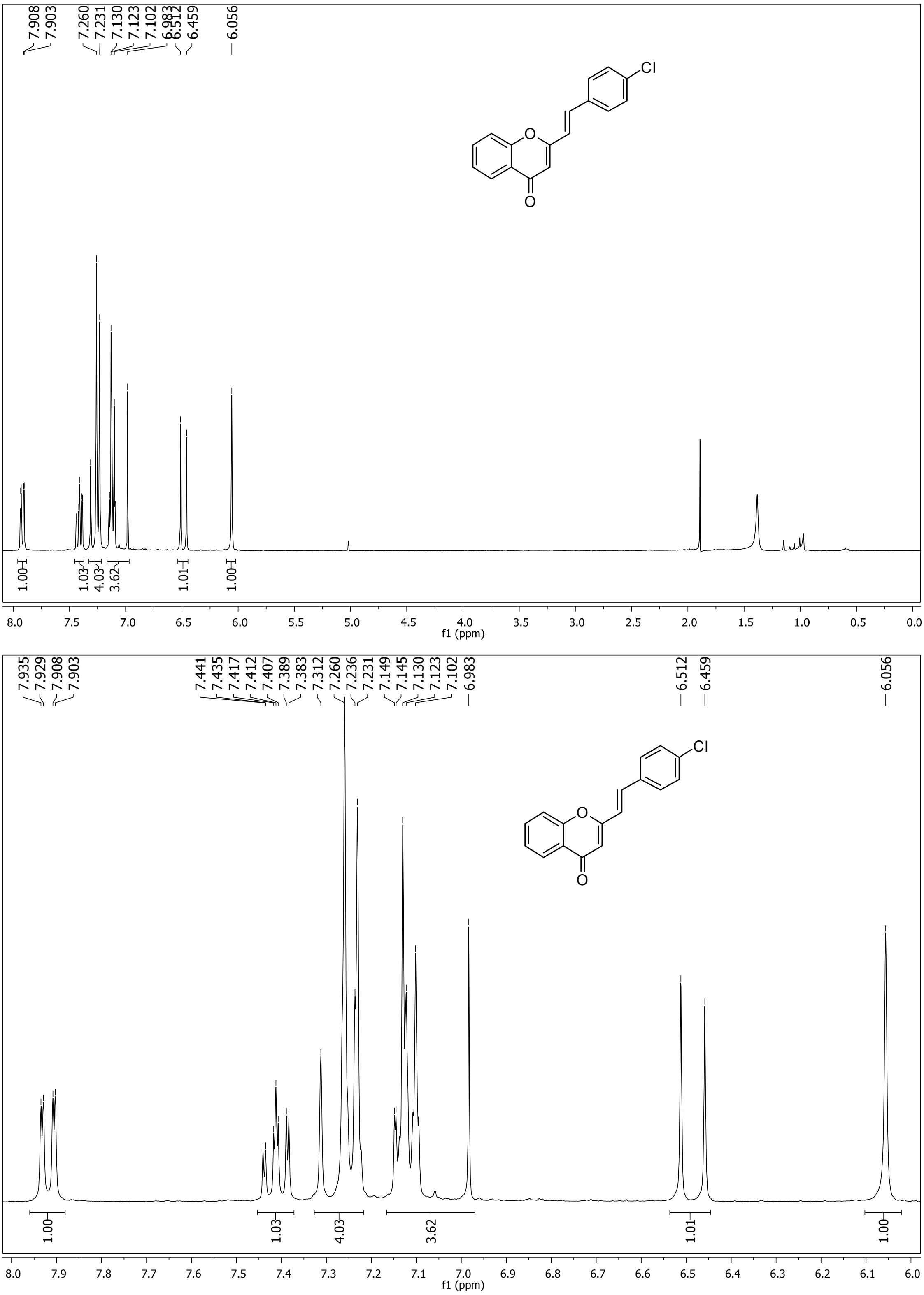

Figure S5. ^{1}H-NMR spectrum of (*E*)-2-[2-(4-chlorophenyl)vinyl]-4*H*-chromen-4-one **5c** ($CDCl_3$, 300.13 MHz).

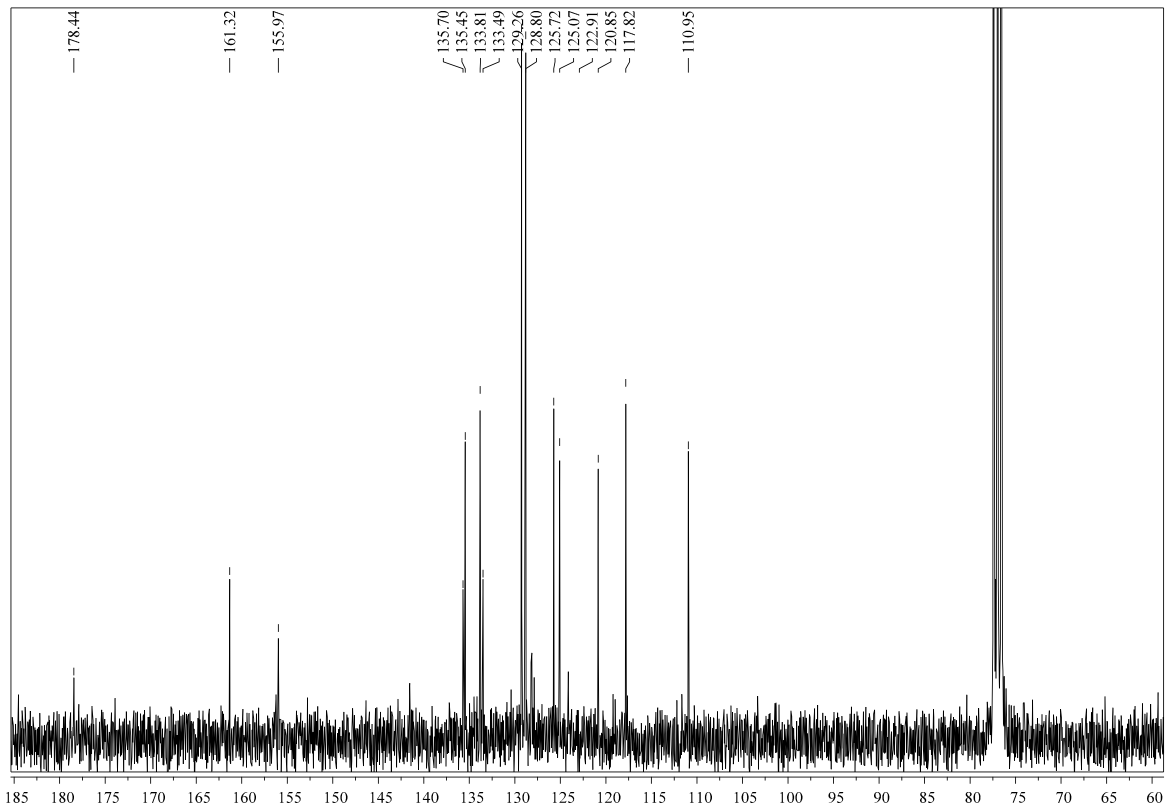

Figure S6. ^{13}C-NMR spectrum of (*E*)-2-[2-(4-chlorophenyl)vinyl]-4*H*-chromen-4-one **5c** ($CDCl_3$, 75.47 MHz).

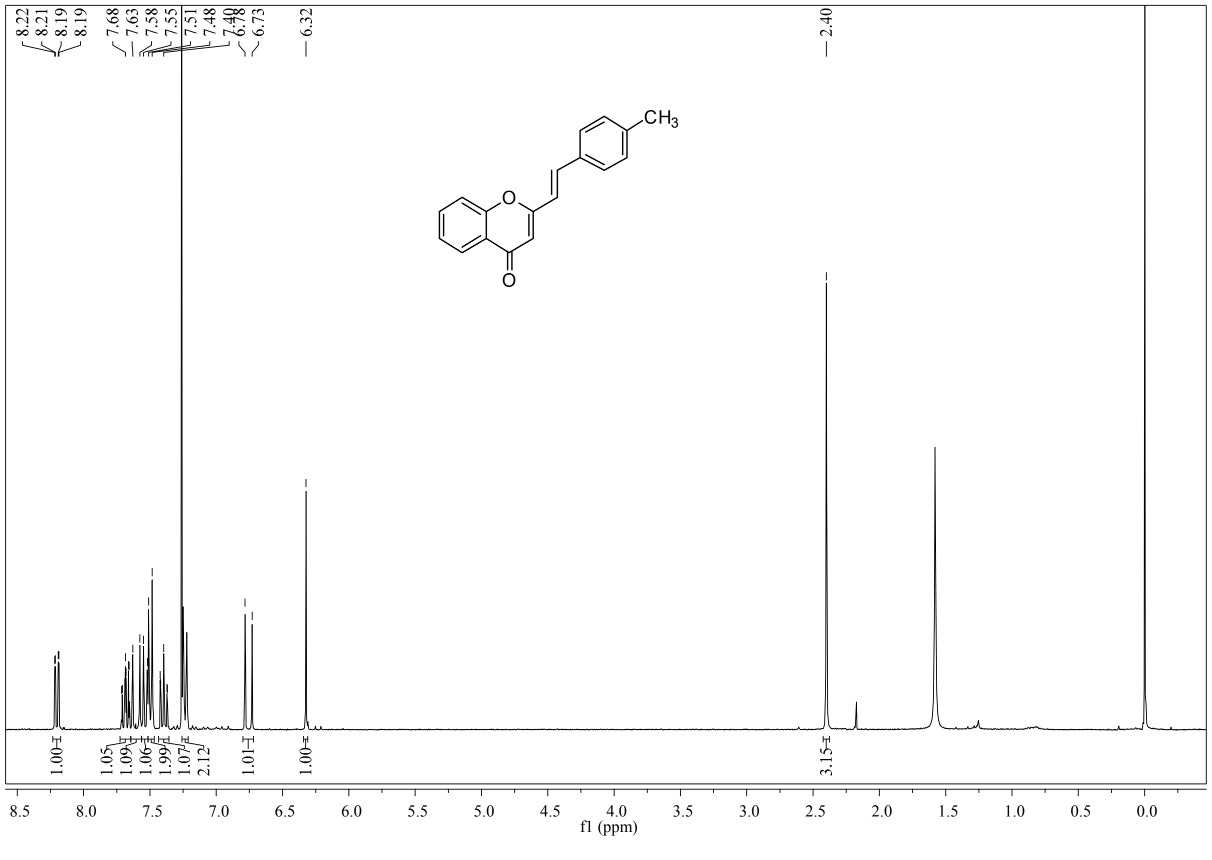

Figure S7. *Cont.*

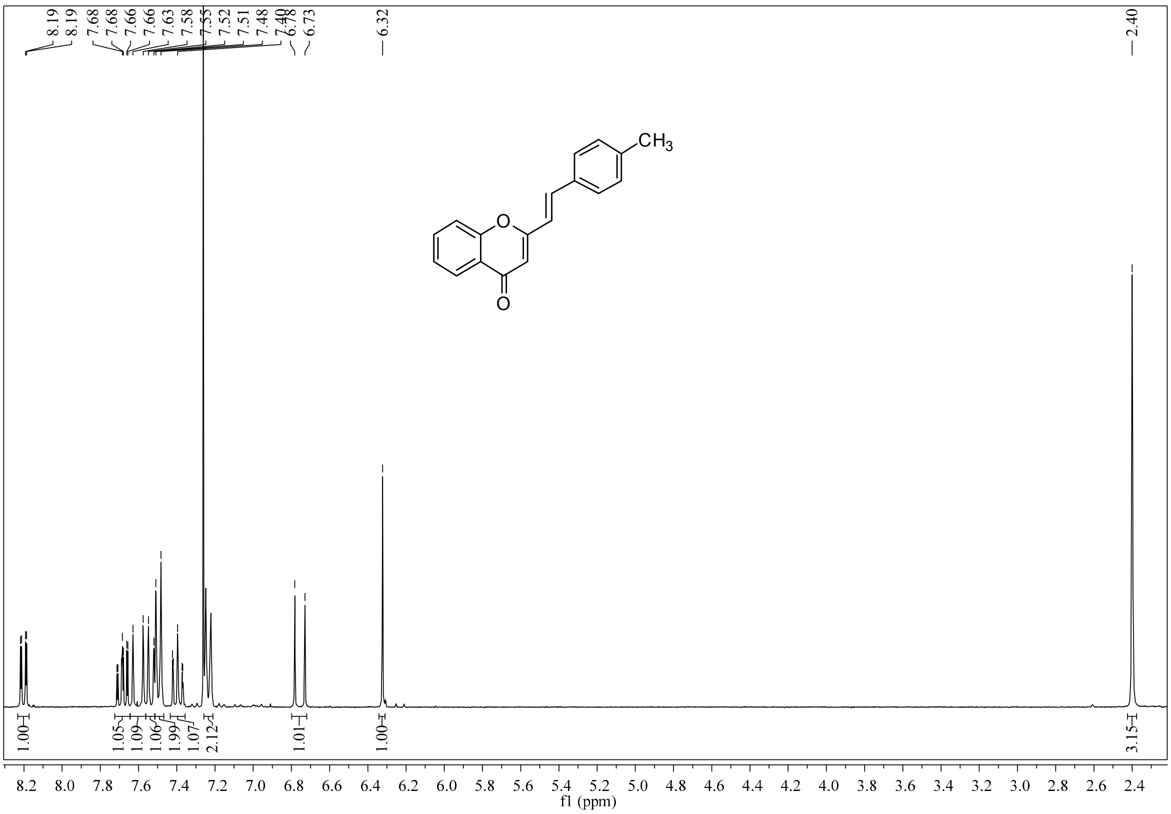

Figure S7. ^{1}H-NMR spectrum of (*E*)-2-[2-(4-methylphenyl)vinyl]-4*H*-chromen-4-one **5d** ($CDCl_3$, 300.13 MHz).

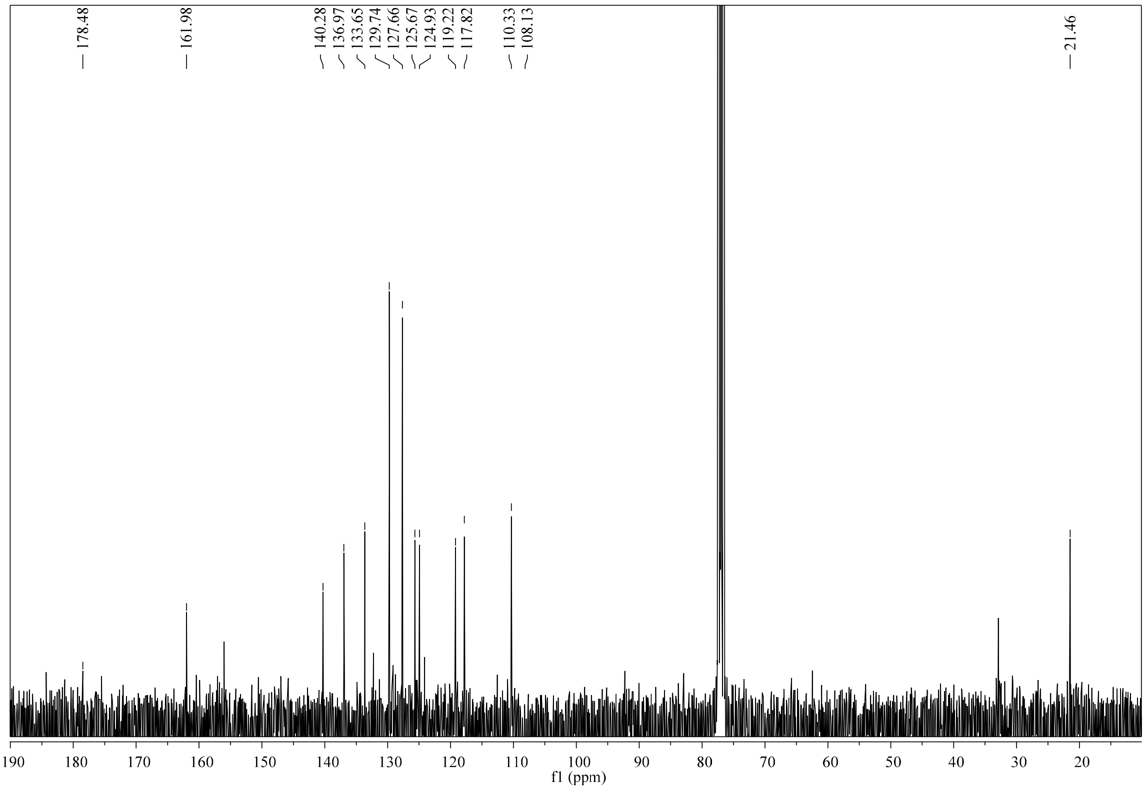

Figure S8. ^{13}C-NMR spectrum of (*E*)-2-[2-(4-methylphenyl)vinyl]-4*H*-chromen-4-one **5d** ($CDCl_3$, 75.47 MHz).

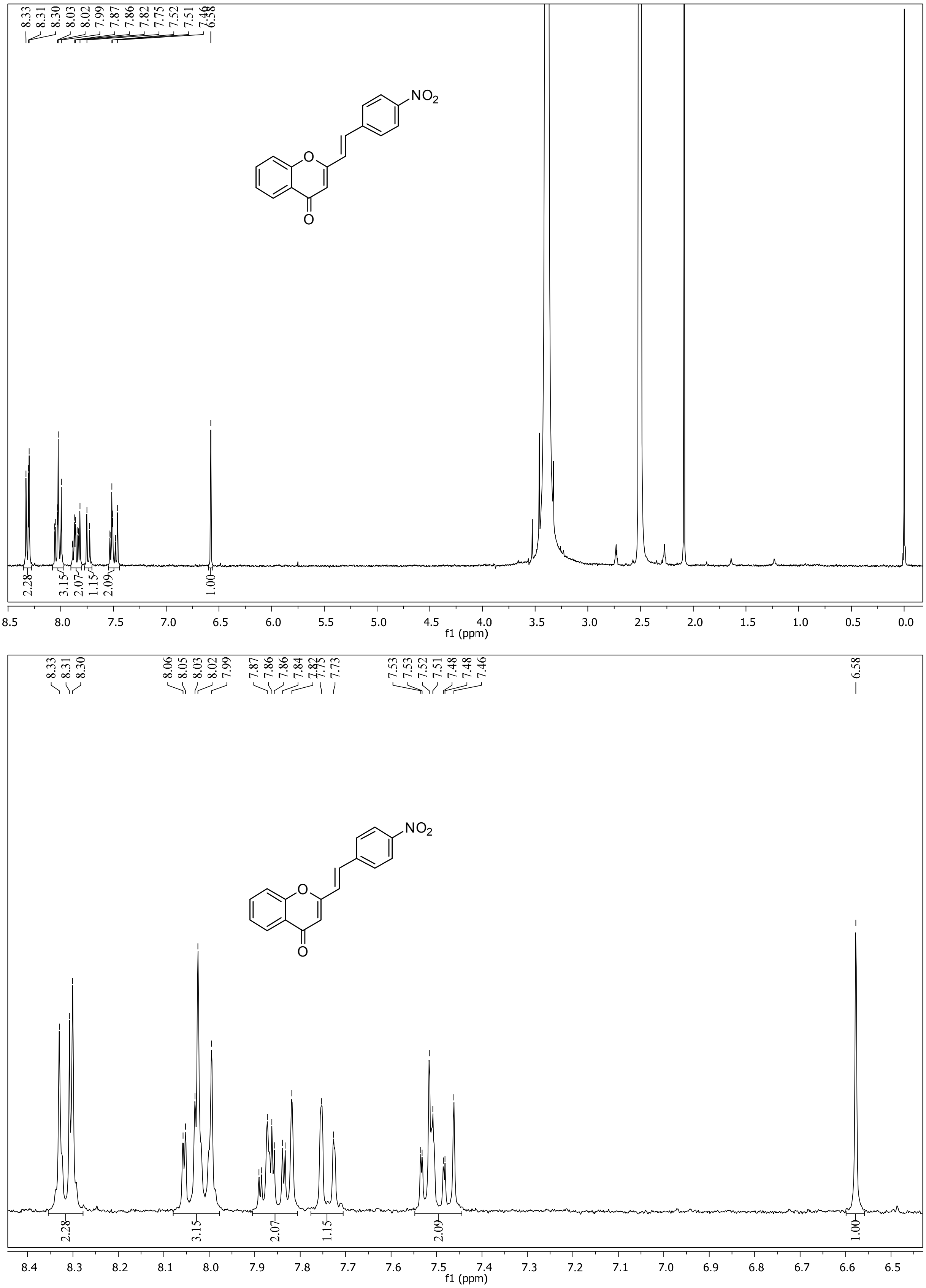

Figure S9. ^{1}H-NMR spectrum of (*E*)-2-[2-(4-nitrophenyl)vinyl]-4*H*-chromen-4-one **5e** (DMSO-*d*$_6$, 300.13 MHz).

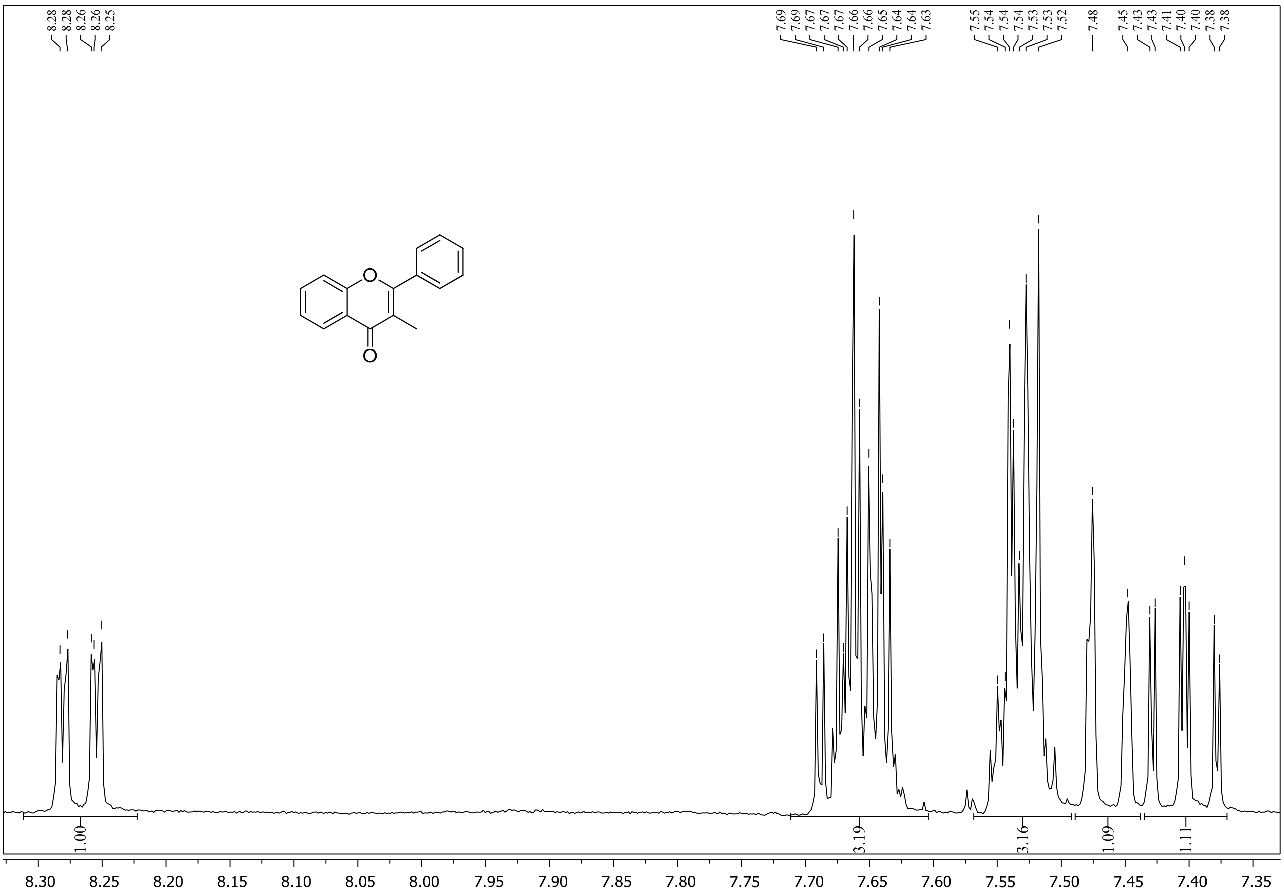

Figure S10. ^{1}H-NMR spectrum of 3-methylflavone **7a** ($CDCl_3$, 300.13 MHz).

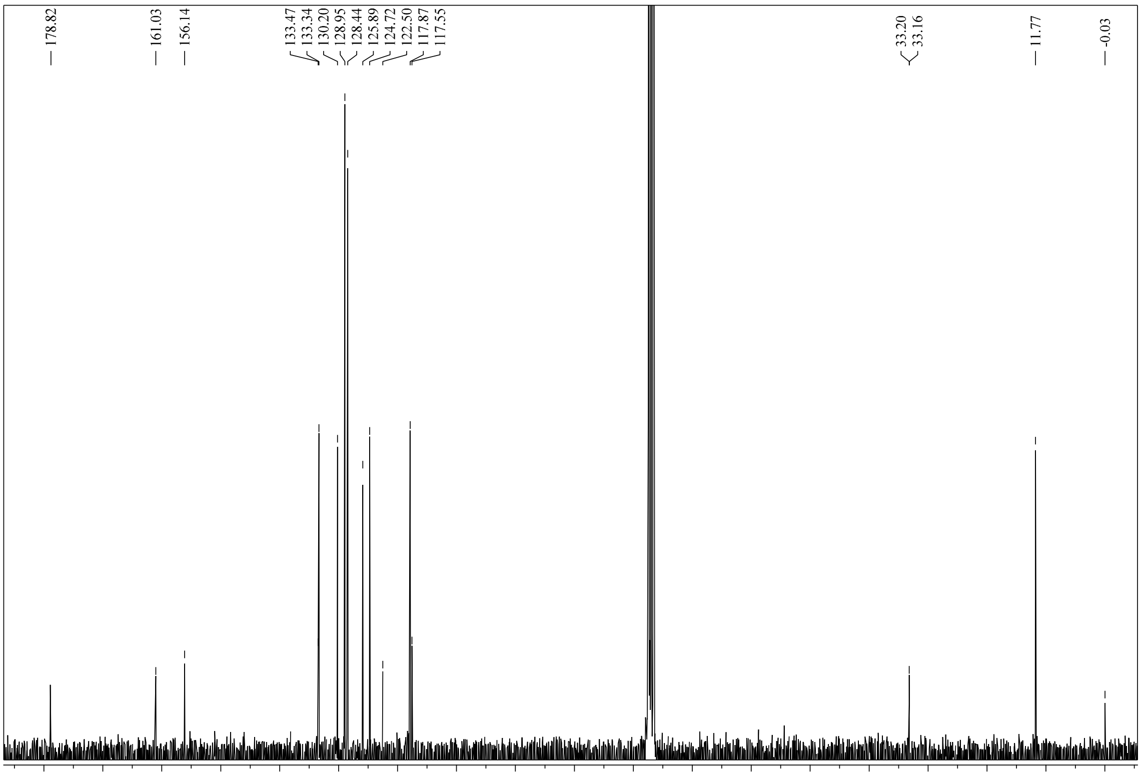

Figure S11. ^{13}C-NMR spectrum of 3-methylflavone **7a** ($CDCl_3$, 75.47 MHz).

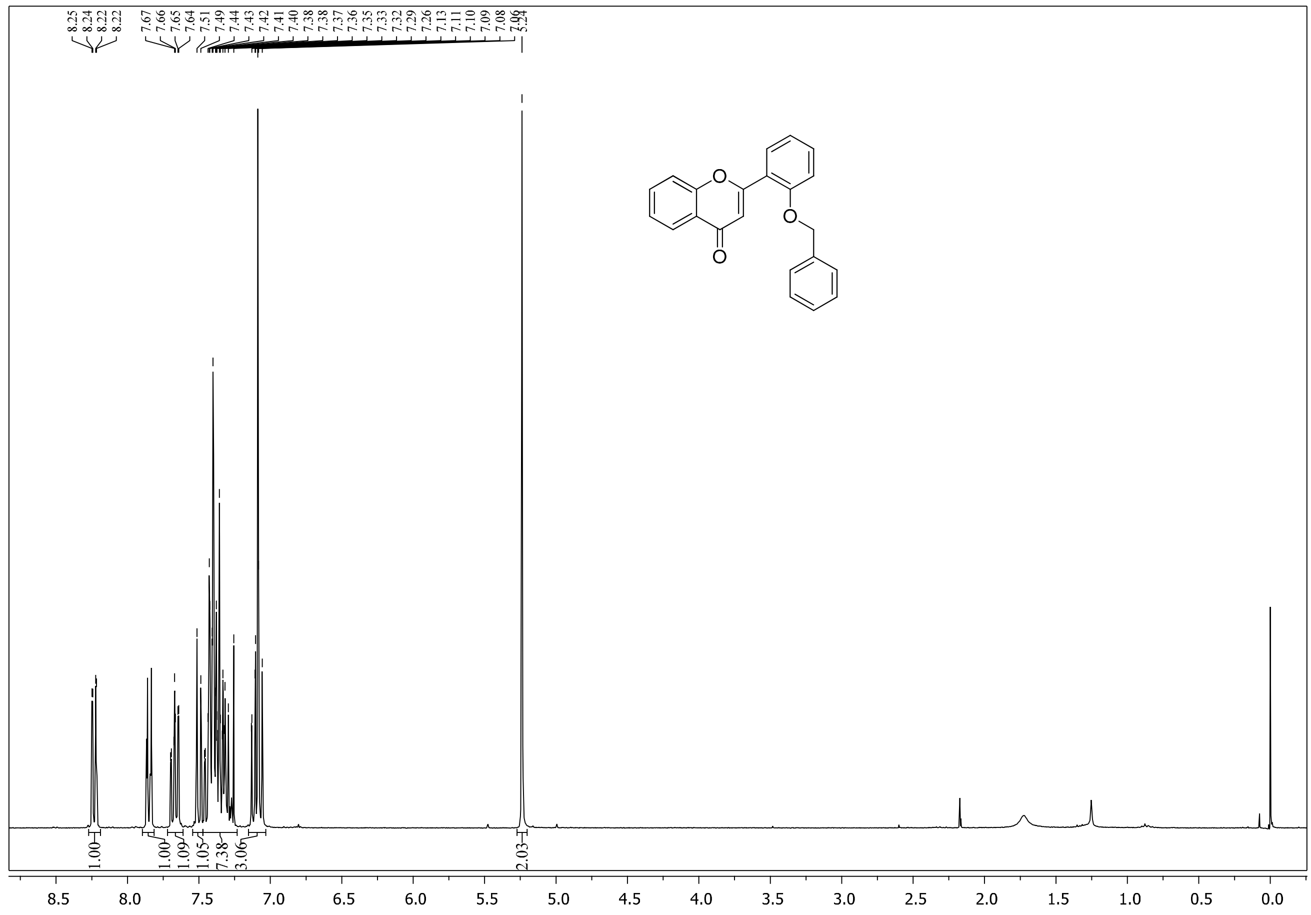

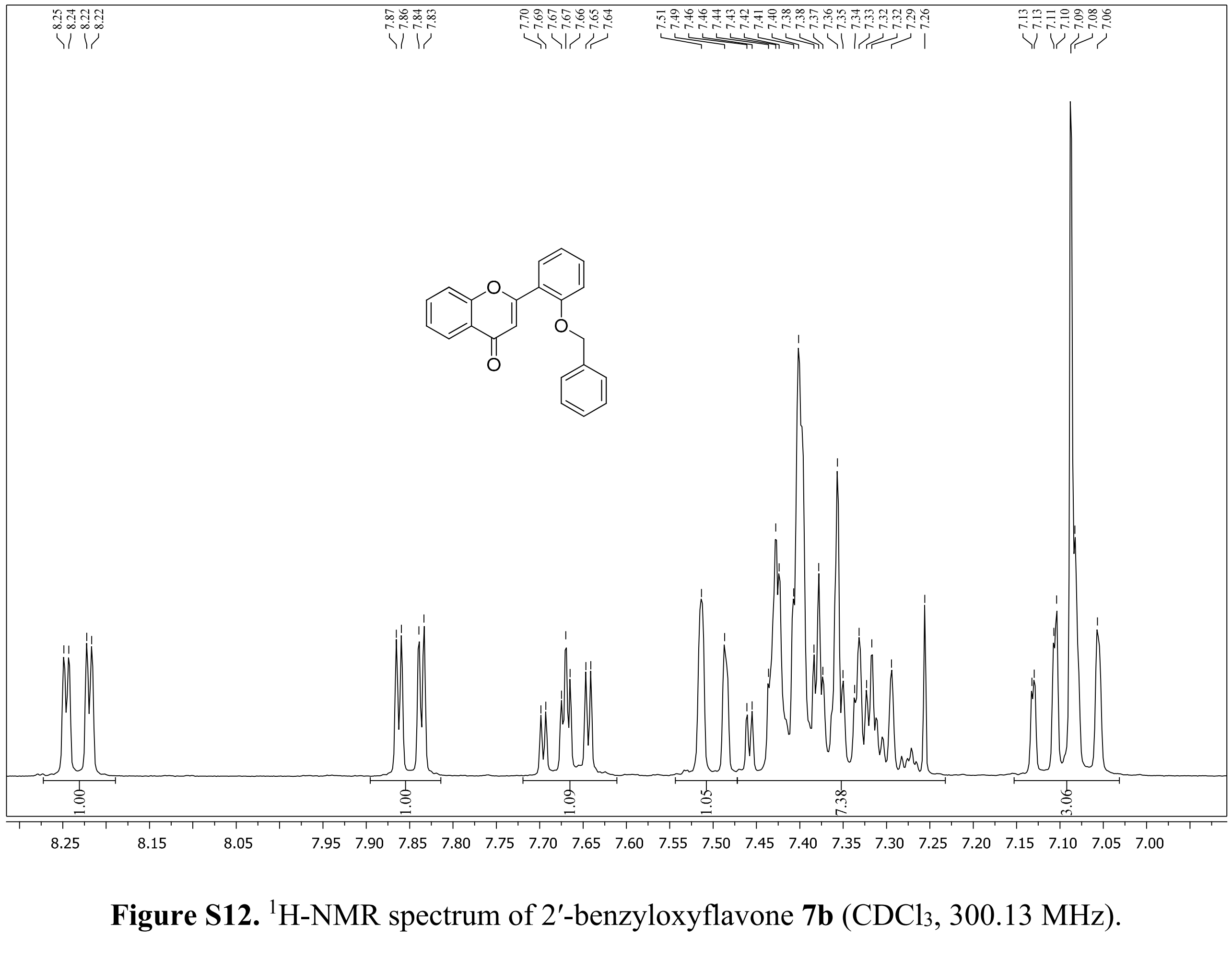

Figure S12. ^{1}H-NMR spectrum of 2′-benzyloxyflavone **7b** ($CDCl_3$, 300.13 MHz).

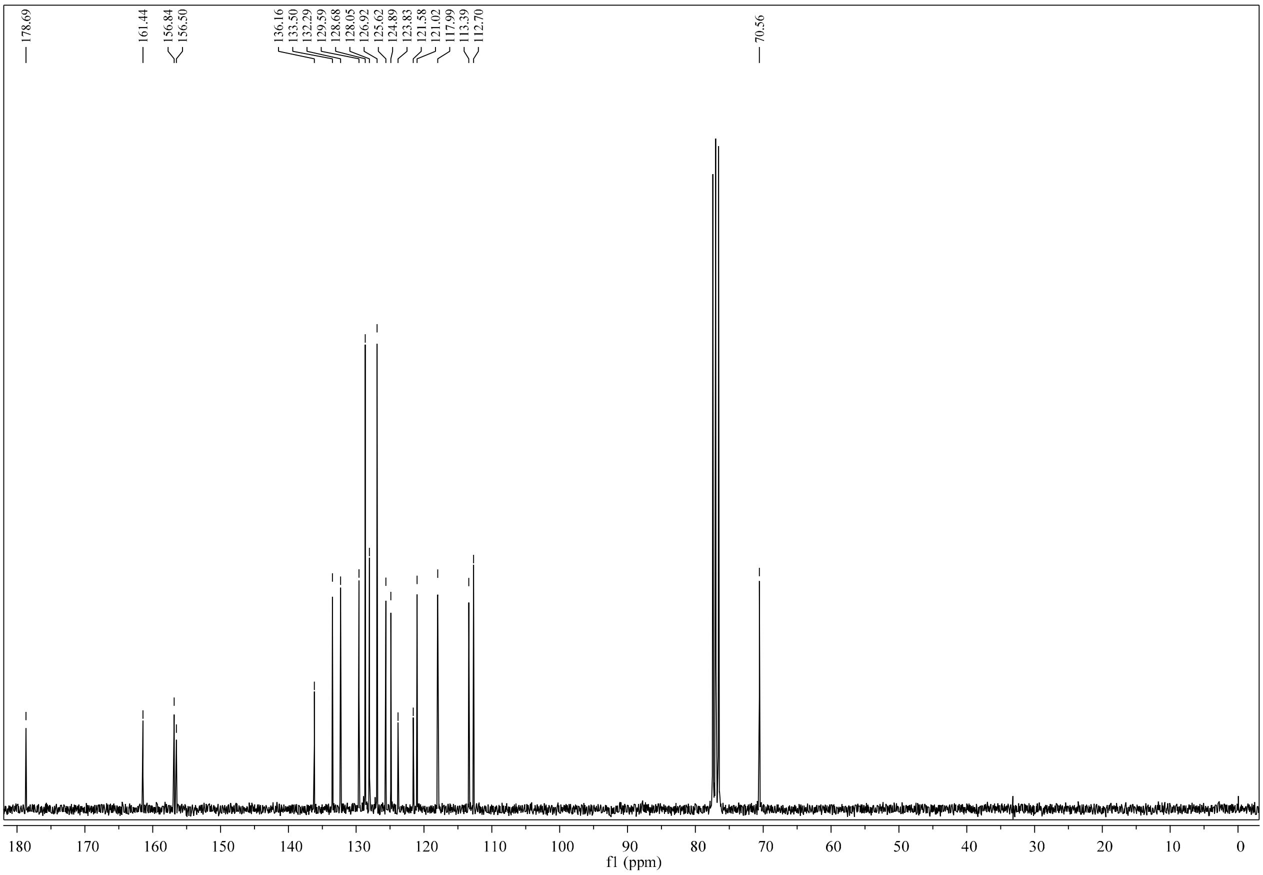

Figure S13. ^{13}C-NMR spectrum of 2′-benzyloxyflavone **7b** ($CDCl_3$, 75.47 MHz).

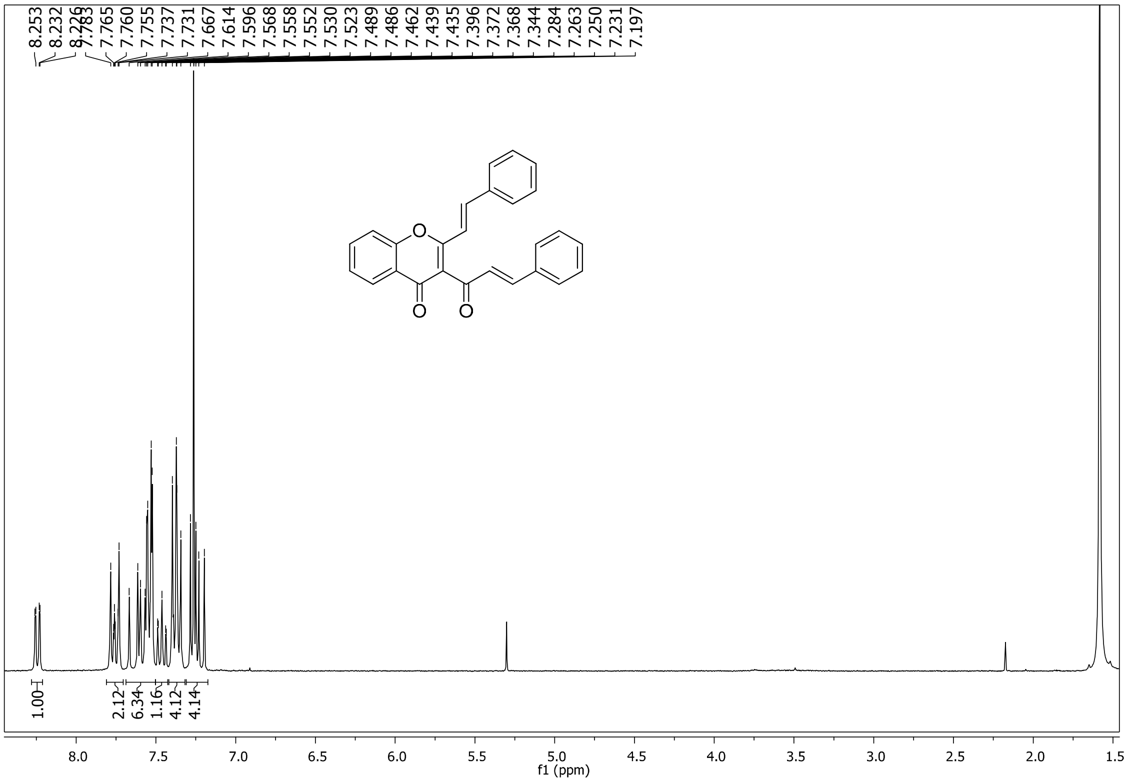

Figure S14. *Cont.*

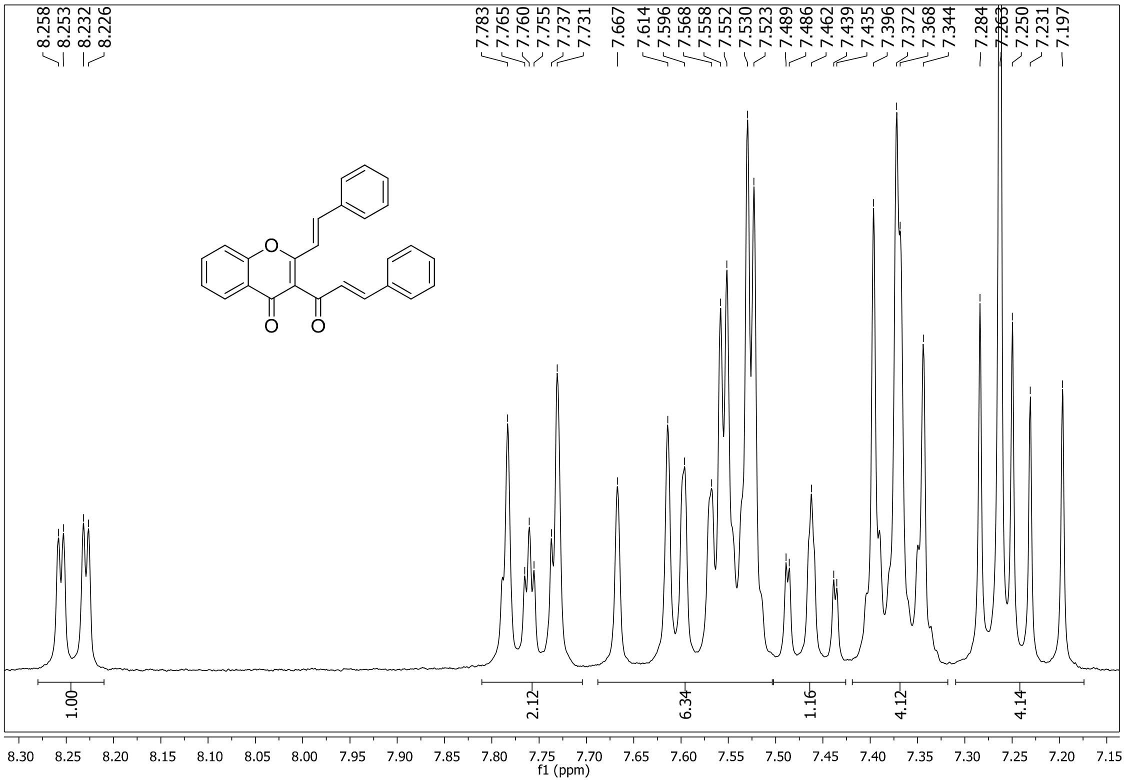

Figure S14. ^{1}H-NMR spectrum of 3-cinnamoyl-2-((*E*)-styryl)-4*H*-chromen-4-one **8** ($CDCl_3$, 300.13 MHz).
